# The zebrafish as a model for studying neuroblastoma

**DOI:** 10.1186/s12935-016-0360-z

**Published:** 2016-11-03

**Authors:** Diana Corallo, Simona Candiani, Michela Ori, Sanja Aveic, Gian Paolo Tonini

**Affiliations:** 1 Neuroblastoma Laboratory, Pediatric Research Institute, Città della Speranza, 35127 Padua, Italy; 2 Department of Earth, Environmental and Life Sciences, (DISTAV), University of Genova, C.so Europa 26, 16132 Genoa, Italy; 3 Unit of Cell and Developmental Biology, Department of Biology, University of Pisa, S.S.12 Abetone e Brennero 4, 56127 Pisa, Italy

**Keywords:** Neuroblastoma, Zebrafish, Embryonic development, Neural crest, Sympathoadrenal cells, Chromaffin cells, Peripheral sympathetic nervous system

## Abstract

Neuroblastoma is a tumor arising in the peripheral sympathetic nervous system and is the most common cancer in childhood. Since most of the cellular and molecular mechanisms underlying neuroblastoma onset and progression remain unknown, the generation of new in vivo models might be appropriate to better dissect the peripheral sympathetic nervous system development in both physiological and disease states. This review is focused on the use of zebrafish as a suitable and innovative model to study neuroblastoma development. Here, we briefly summarize the current knowledge about zebrafish peripheral sympathetic nervous system formation, focusing on key genes and cellular pathways that play a crucial role in the differentiation of sympathetic neurons during embryonic development. In addition, we include examples of how genetic changes known to be associated with aggressive neuroblastoma can mimic this malignancy in zebrafish. Thus, we note the value of the zebrafish model in the field of neuroblastoma research, showing how it can improve our current knowledge about genes and biological pathways that contribute to malignant transformation and progression during embryonic life.

## Background

Neuroblastoma (NB) is the most common extracranial solid tumor arising in the peripheral sympathetic nervous system (PSNS), including spinal sympathetic ganglia and the adrenal medulla [1]. This devastating tumor originates from the sympathoadrenal cell lineage deriving from neural crest cells (NCCs) and is responsible for approximately 15% of childhood cancer-related mortality [2]. NB is characterized by great genetic and biological heterogeneity, and many studies have been performed to understand the complex mechanisms underlying NB origin and progression.

Over the past years, zebrafish has become an attractive alternative tool to the classical mouse model for studying many human diseases, spanning from pediatric to adult tumors and from blood to neurodegenerative disorders (for extended reviews, see [3, 4]). Notably, several experimental techniques that are applied to mice have been extended to zebrafish, and the zebrafish model promises to be extremely useful for development of effective therapeutic strategies for cancer research. For example, zebrafish has emerged as an important tool for the identification of new chemical compounds with potential clinical implications [5], permitting a high-throughput screening of currently available chemical libraries [6].

It is important to highlight that a complete understanding of the biological processes underlying zebrafish PSNS development will be useful for defining the molecular pathways contributing to malignant transformation and NB progression. The biological information obtained with the zebrafish in vivo model might contribute to the discovery of genes and molecular pathways that are dysfunctional during the early stages of NB development.

## Neuroblastoma overview

NB presents as either a localized or metastatic disease, and patients with NB have varying survival rates. Patients with a localized tumor have a good outcome, with an overall survival (OS) of more than 99% 5 years after diagnosis. In contrast, the OS of children older than one year with a metastatic tumor is less than 40% 5 years after diagnosis [7, 8].

A large assortment of copy number variations (CNVs), ranging from numerical CNVs in localized tumors to structural CNVs in metastatic tumors, has been observed in NB. The most frequently observed structural CNVs are loss of chromosomes 1p, 3p, 4p, 9p, 11q and 14q and gain of chromosomes 2p and 17q [9–11]. In the last decade, many efforts have been made to identify candidate NB genes. In fact, *MYCN* oncogene amplification, which is observed in 20% of patients with NB, plays a role in tumor progression and aggressiveness [12]. The genomic amplification of *MYCN* is frequently observed in patients who are resistant to any therapy and is currently a prognostic marker of risk stratification in patients with NB [13]. Other genes have been identified for NB predisposition. *ALK* (anaplastic lymphoma kinase) was the first gene to be identified as a familial NB predisposition gene [14]. Several activating mutations have been discovered in the *ALK* tyrosine kinase domain, although mutations F1174L and R1275Q are the most frequently observed and are associated with a more negative prognosis [15, 16]. Moreover, *LMO1* [17], *BARD1* [10] and *LINC00340* [18] have been identified by genome-wide association studies as other NB predisposition genes. More recently, a massive whole-genome sequencing approach has been used to discover novel key mutations in genes involved in NB onset and progression. Pugh et al. [19] and Molenaar et al. [20] screened more than 200 cases of NB at different clinical stages of the disease and found a low frequency of recurrent mutations. Thus, it seems that NB originates during embryonic life as result of chromosomal instability rather than from a mutation in a single NB gene. There is a general agreement that *MYCN* gene amplification plays a major role in driving NB tumorigenesis, while *ALK* gene mutation cooperates with *MYCN* to enforce tumor aggressiveness [21, 22].

### The in vivo models of neuroblastoma

Previous studies have established different in vivo models of NB. Mouse models of NB are clinically relevant tools for studying the growth and metastasis of this aggressive malignancy. Orthotopic and subcutaneous xenograft mouse models have been extensively generated for the preclinical testing of new therapeutic strategies against NB [23–30]. Notably, the xenograft mouse model displays biological features that limit the possibility of obtaining a standardized in vivo system reflecting tumor pathogenesis. These variables include the manipulation of tumor cells prior to their engraftment outside of the natural tumor microenvironment. Due to the imposed and artificial conditions, xenotransplanted mice frequently develop tumors that do not represent the corresponding human cancer that develops in its native environment [31]. Tumor cell manipulation might result in genome and/or transcriptomic changes distinct from those arising in patients’ cancer cells [31]. To overcome these limitations, Weiss and colleagues [32] generated a transgenic NB mouse model through the overexpression of the human *MYCN* oncogene in neural crest-derived cells driven by the rat tyrosine hydroxylase promoter. These mice spontaneously develop NB exclusively in the sympathoadrenal system with high resemblance to human tumors, demonstrating the involvement of *MYCN* in NB genesis [33–37].

Other transgenic mouse models have helped to elucidate the cellular mechanisms of NB pathogenesis. In addition to *MYCN* gene amplification, the in vivo forced expression of genes that are normally involved in sympathoadrenal development during embryonic life (such as *ALK* and *LIN28B)* drives NB formation [38, 39]. Thus, these animal models support the prenatal origin of this pediatric cancer. Among the increasing number of models dedicated to recapitulating NB origin, the zebrafish model has emerged as a novel important platform for performing in vivo studies of NB pathogenesis. Zebrafish displays several logistic advantages that make it an attractive alternative for mimicking human diseases: (i) its characteristics of reproduction and transparency will allow the study of the first stages of development in a short amount of time; (ii) the optical transparency of zebrafish embryos and larvae will allow the study of the behavior of engrafted human cancer cells or the expression of fluorescently tagged oncogenes; (iii) its morphology will allow the study of disease phenotypes by following the morphological aberrations that often arise in a short amount of time; (iv) it is possible to have thousands of zebrafish embryos and larvae for tissue specimen collection and high-throughput drug screening; (v) an increasing number of technologies are available for zebrafish genetic manipulation, such as transient/stable gene loss and gain of function as well as high-throughput DNA and RNA sequencing techniques. Thus, the zebrafish model is a powerful tool for dissecting the molecular pathways involved in PSNS development and NB origin. This biological information is important not only for understanding exactly when and where malignant transformations occur but also for more the rapid determination of possible genetic or chemical tumor progression modifiers that might guarantee novel and more effective therapies.

## Zebrafish as a model for studying peripheral sympathetic nervous system development

Increasing evidence indicates that NB arises from the NCC lineage during embryonic life (for an extended review, see [40]); thus, it is crucial to understand the characteristics of NCCs under both normal and pathological conditions in order to dissect the initial tumorigenesis steps of this embryonic cancer.

The cells of the sympathetic ganglia and mature adrenal medulla originate from a transient pool of NCCs. These cells pass through four critical developmental phases. First, NCCs undergo an epithelial to mesenchymal transition (EMT) from the most dorsal region of the neural tube and differentiate into sympathoadrenal precursors. Then, sympathoadrenal precursors migrate toward the ventral portion of the notochord near the dorsal aorta. Finally, these cells activate the neuronal differentiation program, giving rise to the mature sympathetic ganglia and chromaffin cells of the adrenal medulla.

### Induction of an epithelial to mesenchymal transition in neural crest cells

After neurulation, NCCs undergo an EMT from the roof plate of the neural tube, delaminating from the neuroepithelium and then migrating through the periphery, where they differentiate into several cell types, including cells of the peripheral nervous system and enteric nervous system, pigmented cells, Schwann cells, adrenal medullary cells, and cartilage cells of the craniofacial skeleton [41–43]. The downregulation of cadherins on the cell surface (usually mediated by the upregulation of mesenchymal transcription factors, such as Snail, Slug and Twist), cytoskeleton remodeling and the synthesis of proteases are key events that mediate this transition. These cellular changes are also essential for the development of many tissues during embryogenesis. Interestingly, similar modifications are recapitulated during pathological processes, such as fibrosis, cancer and drug resistance [44, 45].

Recently, in vitro studies have suggested that an EMT might also be involved in NB progression, and Nozato and colleagues [46] found that *KRT19*, *ERBB3*, *TWIST1* and *TCF3* EMT-related genes are differentially expressed in a cohort of patients with NB. Thus, in addition to *MYCN* gene amplification, the expression level of EMT-related genes might become an additional prognostic marker for patients with NB. Despite these recent discoveries, there is no solid evidence from studies using in vivo models that supports the relationship between EMT and NB pathogenesis. Therefore, additional studies will be necessary to elucidate whether EMT plays a role in tumor development and eventual metastasis in NB. Consequently, the window of time in which EMT impairment might lead to the modulation of tumor-associated signals should be defined.

### Neural crest cell migration and differentiation

**Fig. 1:**
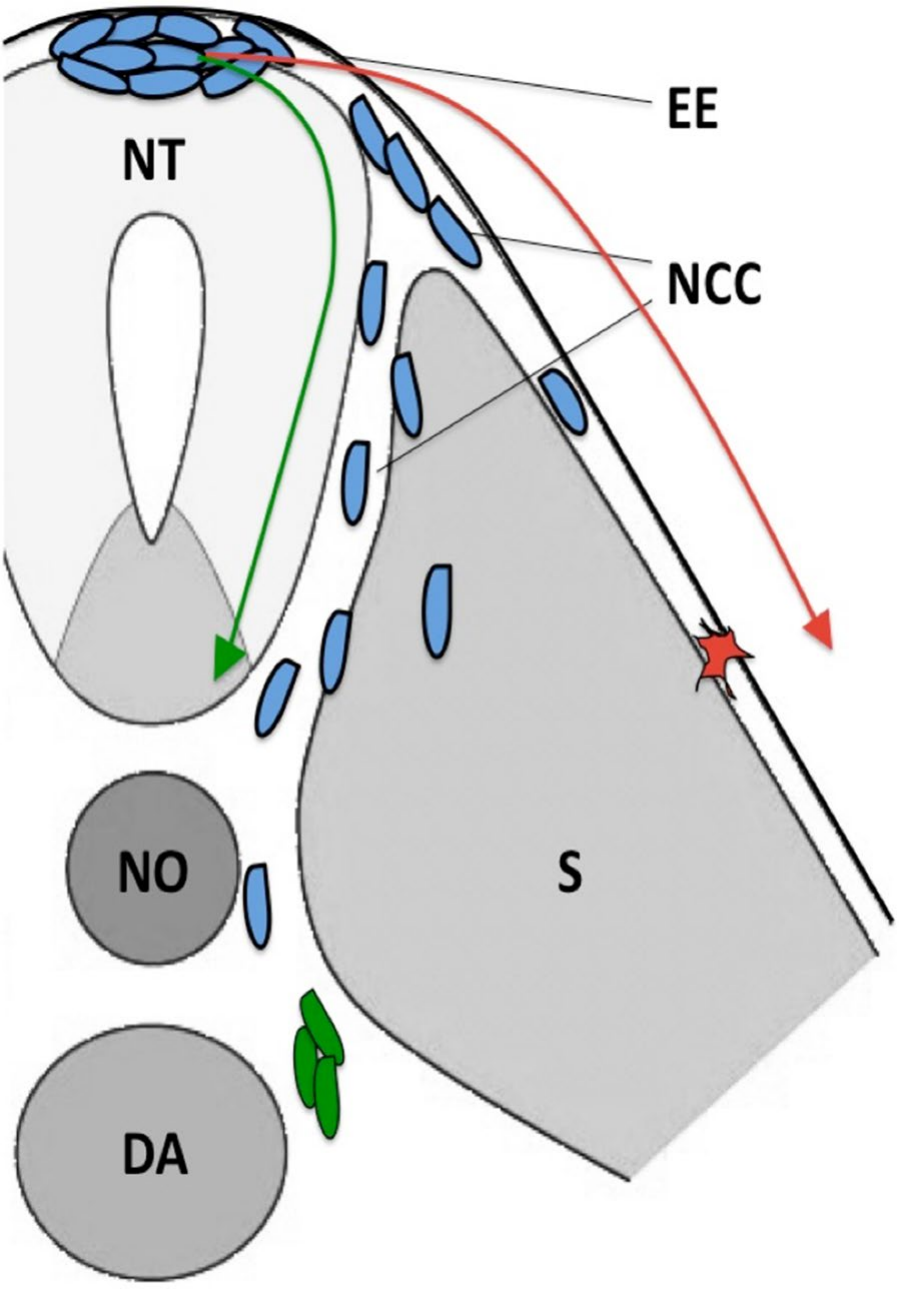
Patterns of NCC migration in zebrafish. Schematic cartoon depicting the two main migratory pathways of NCCs during embryonic development (transverse section of a vertebrate embryo). NCCs (*blue*) follow the ventromedial pathway (*green arrow*) and migrate between the neural tube and somites and then bilaterally reach the dorsal portion of the dorsal aorta, where they differentiate into sympathoadrenal progenitors (*green*). NCCs that migrate dorsolaterally (*red arrow*) between somites and the overlying ectodermal epithelium give rise to pigment cells (*red*). *NCCs* neural crest cells, *EE* ectodermal epithelium, *NT* neural tube, *NO* notochord, *DA* dorsal aorta, *S* somite

After an EMT begins, NCCs acquire a polarized morphology and follow two migratory pathways away from the neural tube: the dorsolateral and the ventromedial (Fig. 1). NCC-derived sympathoadrenal precursors ventromedially migrate within the sclerotome, whereas the dorsolateral pathway mostly generates the pigmented cell lineage, including melanocytes [47–49]. Nevertheless, in the trunk region, the route of the NCCs and their migration schedule are not perfectly mirrored in all species but rather show importantly dissimilar patterns among them [50]. Spatially and temporally regulated extrinsic signals from the neural tube, somites, notochord and dorsal aorta are essential for the migration and specification of sympathoadrenal precursors. These signals include many permissive factors, such as the extracellular matrix proteins laminins, versican and fibronectin, which drive the earliest migrating sympathoadrenal precursors into the ventromedial pathway [51–53]. In parallel, ephrins, chondroitin sulfate proteoglycans and other inhibitory molecules block the alternative dorsolateral pathway.

### The molecular development of the peripheral sympathetic nervous system in zebrafish

**Fig. 2:**
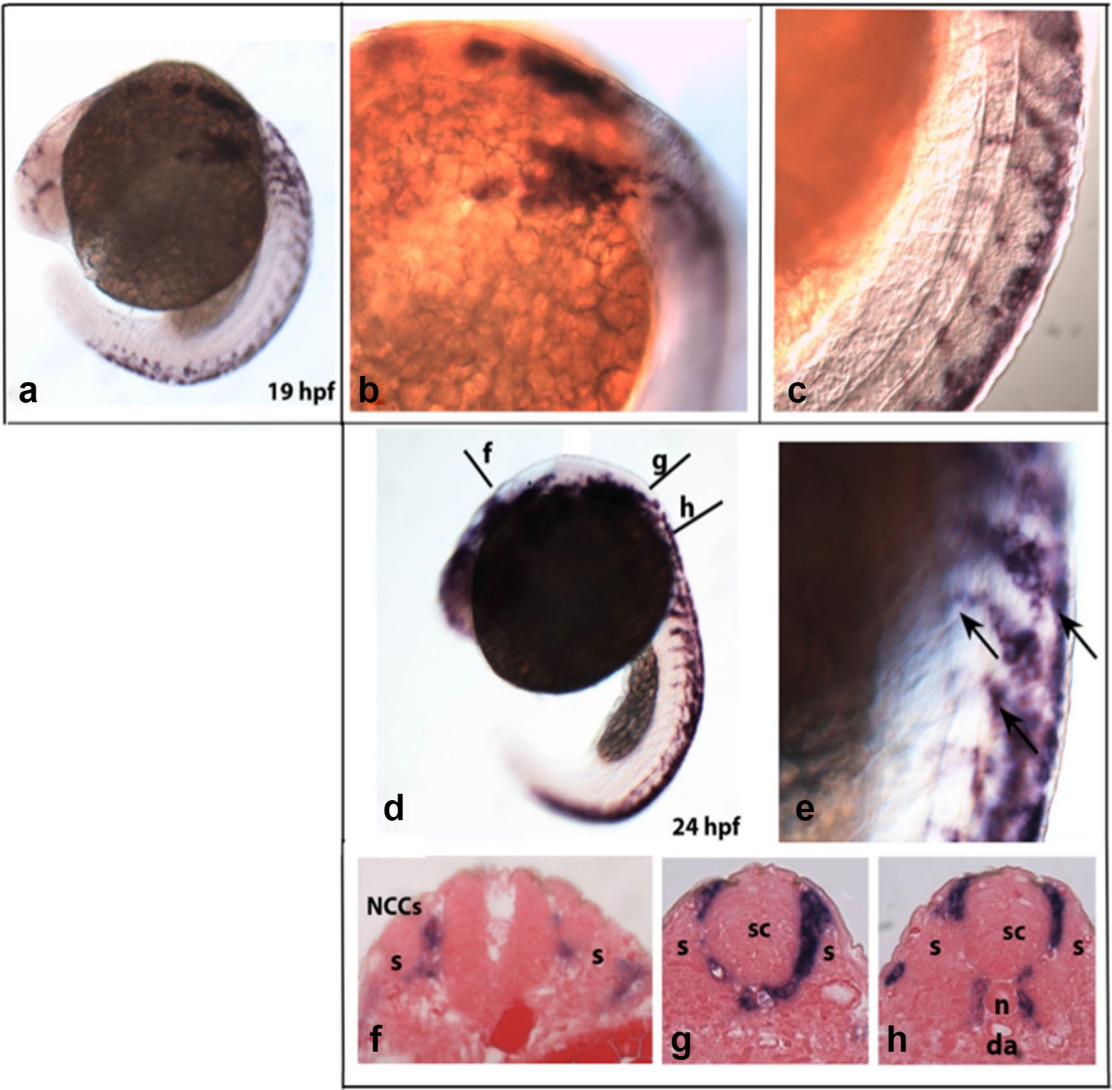
Expression of crestin in neural crest cells (NCCs) during zebrafish development. **a** *Crestin* is expressed in premigratory NCCs in wild-type embryos at 19 hpf. **b**, **c** Higher magnification images of the embryo in **a**, showing the streams of migrating NCCs labeled with the *crestin* probe. **d**, **e** At 24 hpf, trunk migratory NCCs labeled by *crestin* (*arrowheads*) gradually migrated toward the ventral dorsal aorta. **f**–**h** Cross sections through the levels (*f*, *g*, *h*) shown in **d**. Streams of trunk NCCs defined by *crestin* expression migrate between the somite and the neural tube. *da* dorsal aorta; *n* notochord; *s* somite; *sc* spinal cord

The development of the PSNS in zebrafish has been well examined [54, 55]. In fact, despite several structural and physiological differences between zebrafish and mammals, the morphogenesis and differentiation of sympathetic neurons in zebrafish are strongly comparable to those of other vertebrates. In zebrafish, trunk sympathoadrenal precursors begin to migrate approximately 16 h post-fertilization (hpf) from somite 7 and follow the ventromedial pathway, indicating that the formation of sympathetic neurons is similar to that of other vertebrates [54, 55]. NCCs are easily identified by the expression of the transcription factor Sox10 [56, 57], and crestin is a multicopy retro-element expressed in pre-migratory and migratory NCCs (Fig. 2) [58]. Moreover, in mammalian embryos, the receptor tyrosine kinase proto-oncogene ErbB3 and its dimerization partner ErbB2 are required for proper trunk NCC migration. Notably, mice mutated in either ErbB display embryonic lethality, limiting their use within early developmental stages [59, 60]. Honjo and colleagues [61] reported that zebrafish mutants lacking ErbB3 or ErbB2 do not form trunk NC-derived sympathetic neurons, whereas cranial NC derivatives appear normal. These data suggest that ErbB2/ErbB3 signaling is required for trunk NCC migration and localization in the embryo within a specific window of time. Moreover, Sox10 increases the expression of ErbB3, describing a positive activating loop from an intrinsic transcriptional factor that can modulate the migration responsiveness of cells [62–64]. Interestingly, the proto-oncogene *MYCN* appears to be required for the migration, survival and/or differentiation of NCCs that migrate to the dorsal aorta. Accordingly, homozygous mutant mouse embryos display embryonic lethality (at approximately 11.5 days of gestation), and several developing organs are affected, including the dorsal root ganglia and sympathetic ganglia of the PSNS [65–67].

When sympathoadrenal precursors reach the dorsal aorta, other molecules, such as *N*-Cadherin, CXCL12, and Ephrins, play a critical role in the initiation of the segmental organization of sympathetic ganglia in a discrete and metameric pattern [68]. Moreover, the dorsal aorta-derived bone morphogenetic factors (BMPs) are essential for the differentiation of sympathoadrenal precursors into mature sympathoadrenal cells. Later, cells destined to become chromaffin cells of the adrenal medulla retain their responsiveness to BMPs. Consequently, sympathoadrenal precursors continue to migrate toward the BMP4-secreting cortical cells of the adrenal cortex and finally form the adrenal medulla near the anterior pronephros [69]. The sympathoadrenal lineage is specified by a tightly regulated set of transcription factors, and in zebrafish, this process has been characterized in detail. After reaching the proper location near the dorsal aorta, sympathoadrenal precursors begin to express the pan-neuronal marker 16A11, which comprises members of the Hu family of RNA binding proteins [70]. In 2002, An and colleagues [54] demonstrated that sympathoadrenal cells differentiate from the rostral toward the caudal part of the embryo. In fact, cervical neurons develop at 2 days post-fertilization (dpf) and comprise the superior cervical ganglion (SCG), whereas trunk sympathetic neurons express 16A11 several days later (between 4 and 8 dpf). The reason for this delay remains unknown since trunk sympathoadrenal precursors reach the dorsal aorta between 24 and 36 hpf, when the primary source of BMP is still present. Indeed, since NB can develop anywhere along the sympathetic axis beside the adrenal medulla, it may be relevant to investigate how these cells respond differently to BMPs in terms of both space and time [54]. Thus, it is likely that NB may arise from earlier crest derivatives before the development of the sympathoadrenal lineage [1, 71]. This phenomenon could contribute to the heterogeneous histology and pathology found among NBs.

Other transcription factors are required for sympathoadrenal cell development and maintenance, such as *phox2b* and *zash1a*, which in turn activate the expression of *hand2* and later markers, such as *phox2a, GATA2* and *AP*-*2 alpha* (*tfap2a*) [72–75]. For example, the hand2 zebrafish mutant (*hands off*) fails to reach the complete differentiation of sympathoadrenal cells, even though some early PSNS markers are normally expressed [74]. An analogous phenotype has been observed in mutant embryos lacking the tfap2a gene (mount blanc mutants), in which sympathoadrenal cells fail to differentiate completely [76, 77].

**Fig. 3:**
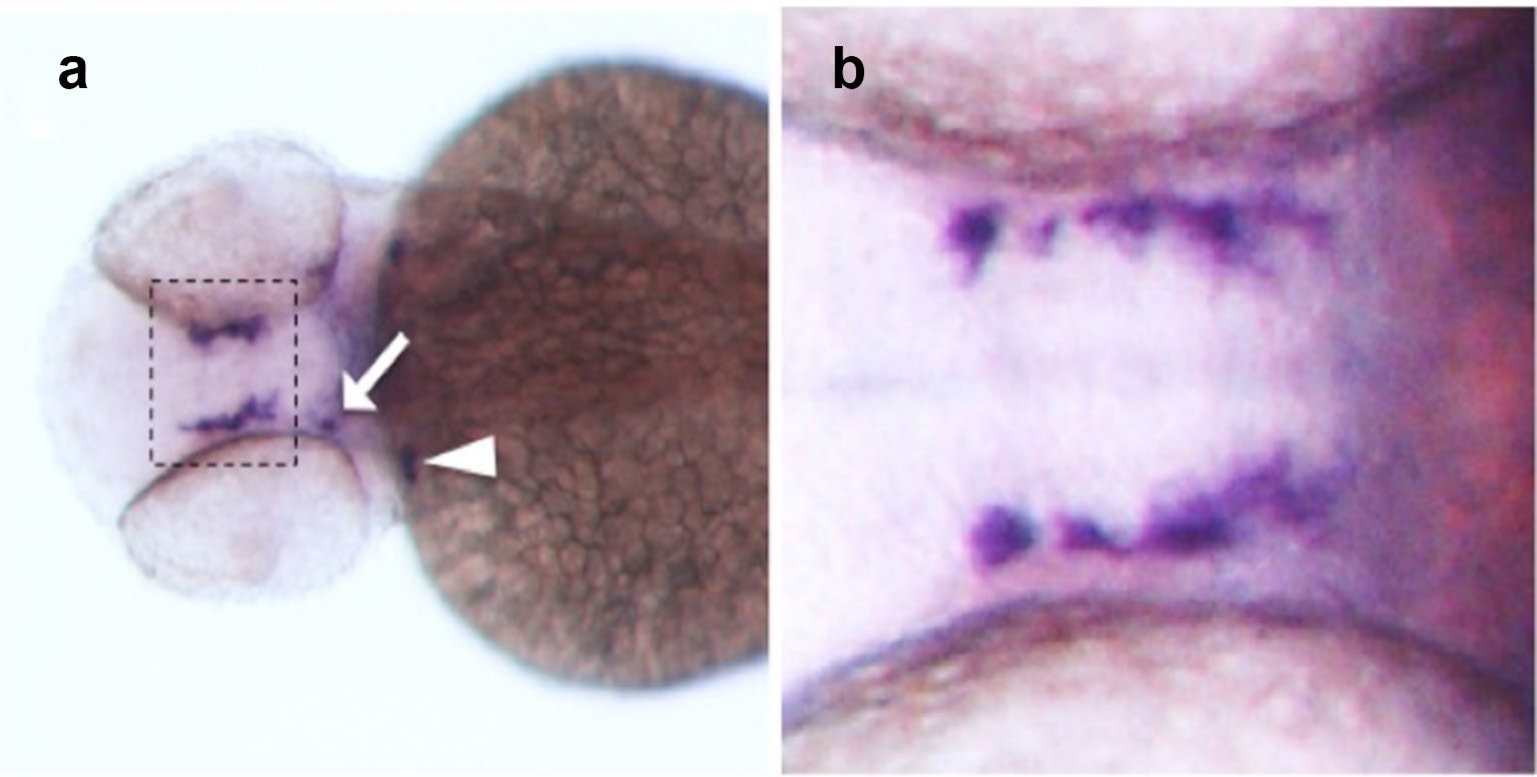
Expression of TH by fully differentiated sympathetic neurons in zebrafish. **a** Dorsal view of a wild-type embryo probed at 48 hpf by in situ hybridization with *TH*. At this developmental stage, *TH* mRNA is expressed by dopaminergic neurons of the ventral diencephalon (*hatched box*), arch-associated catecholaminergic neurons (*arrowhead*) and neurons constituting the locus coeruleus (*arrow*). **b** Higher magnification of dopaminergic neurons stained with the *TH* probe

Finally, sympathoadrenal cells undergo catecholaminergic terminal differentiation for the formation of the mature sympathetic chain and the adrenal gland. This last differentiation step requires the expression of tyrosine hydroxylase (*TH*) and dopamine B-hydroxylase (*DBH*) genes, which are involved in the conversion of the amino acid l-tyrosine to noradrenalin [78]. In zebrafish, both genes are used to detect fully differentiated sympathetic neurons. The TH protein is detectable in the superior cervical ganglion starting from 2 dpf, whereas its expression in trunk sympathetic neurons becomes detectable at 7 dpf (Fig. 3). In general, by 10 dpf, the majority of sympathetic neurons express *TH,* and the complete maturation of sympathetic ganglia and chromaffin cells is reached at 28 dpf [54]. Expression protein analysis of DBH during sympathetic neuron development shows that DBH is detectable 1 day after TH expression [54].

Newly developed high-resolution imaging techniques and genetic tools will continue to provide unique insight into the mechanisms controlling cell differentiation during embryogenesis [79]. Accordingly, the identification of new zebrafish mutants affecting PSNS development might contribute to the in vivo analysis of NB onset.

## Zebrafish genetic models of neuroblastoma

As previously described, several in vivo strategies have been used to dissect the molecular pathways that normally coordinate sympathetic neuron development. In particular, forward and reverse genetic techniques and transgenic zebrafish models have provide new insight into the function of the genes and pathways involved in either normal vertebrate development or human cancer formation.

### The proto-oncogene MYCN: functional interactions with ALK receptor and the tumor suppressor NF1

During normal sympathoadrenal development, the proto-oncogene *MYCN* is highly expressed in the early post-migratory neural crest cells, where it regulates the ventral migration and expansion of NCCs, whereas MYCN protein levels gradually decrease in differentiating sympathetic neurons. This observation suggests that sympathoadrenal maturation requires low or absent MYCN expression [80, 81]. In zebrafish, the persistent expression of the *MYCN* gene in sympathoadrenal precursors, which mimics the amplification observed in more than 20% of patients with NB, dramatically blocks development toward a chromaffin-like cell phenotype, leading to NB mass formation in the interrenal gland [21, 22]. The tumor formation in the zebrafish interrenal gland recapitulates the adrenal medullary site of origin that is observed in approximately half of the children affected by NB [82]. In contrast, tumors in the murine MYCN transgenic model arise predominately in the sympathetic cervical ganglia complex and the superior cervical ganglia [80, 83]. Tumor masses arising in MYCN-overexpressing zebrafish are histologically, immunohistochemically and ultrastructurally very similar to those arising in humans, supporting the use of this model to investigate NB tumorigenesis. In addition, both the onset and penetrance of this disease are markedly enhanced by the co-expression of the transgene encoding the activated ALK receptor tyrosine kinase, carrying the most common and aggressive activating mutation, F1174L, found in humans [84]. This mutation, although not tumorigenic itself in zebrafish, potentiates the oncogenic power of *MYCN* by blocking the apoptosis of MYCN-overexpressing sympathoadrenal neuroblasts of the interrenal gland [21]. In fact, this double-transgenic animal model allowed the elucidation of the molecular mechanisms through which *MYCN* and *ALK* cooperate in the generation of aggressive tumors. Additional studies using the zebrafish model will be required to determine whether other mutational events can induce NB in a synergistic way, as in the MYCN-ALK model.

More recently, the zebrafish MYCN transgenic model has been used to study which domain of the tumor suppressor protein NF1 is important for inhibiting the growth of NB [85]. When He et al. deleted the gene for the zebrafish version of NF1, the penetrance and the disease onset of MYCN-induced NBs were accelerated and associated with the aberrant activation of the RAS-MAPK signaling. Interestingly, supplying zebrafish with the GTPase-activating protein (GAP)-related domain (GRD) of NF1 was enough to suppress NB growth. In addition, the MEK inhibitor against the RAS-MAPK pathway, trametinib, synergizes with, 13-*cis* retinoic acid, to suppress *nf1*-deficient neuroblastoma in vivo. These findings establish the double-transgenic line *nf1*-deficient/MYCN-overexpressing zebrafish as a valuable tool for testing new therapeutic strategies for NB with mutations affecting the RAS-MAPK pathway, which appear to be a major cause of relapse in patients with NB [85].

### The homeobox transcription factor PHOX2B

Previous in vivo models have established PHOX2B as a key regulator of autonomic neuron development. In fact, PHOX2B mutant mice showed embryonic lethality due to a failure of PSNS formation [86]. Moreover, TH-MYCN transgenic mice developed NB in early postnatal sympathetic ganglia predominantly composed of PHOX2B-positive neuronal progenitors [83], and heterozygous germline mutations of PHOX2B are associated with a subset of familial NB [87–90]. These observations indicate a central role of PHOX2B in predisposition to NB.

In 2013, Pei and collaborators [91] reported that morpholino-mediated PHOX2B deletion in zebrafish leads to an impaired sympathetic neuronal differentiation of the PSNS. Similarly, the overexpression of an NB-linked truncation mutation (K155X) [90] or a frameshift mutation (676delG) [85] in the presence of endogenous PHOX2B leads to a similar block in PSNS differentiation, indicating that these variants function in a dominant-negative manner [91]. This study presents an embryonic in vivo model in which aberrant PHOX2B functioning through either allelic deletion or dominant-negative mutations promotes an impaired differentiation of sympathetic neuronal progenitor cells. Future experiments will investigate in more detail the regulatory relationships among the mentioned mutations related to NB.

### Aberrant expression of the fgf8 gene in Hagoromo mutants

The histological screening of a collection of zebrafish insertional mutants revealed that four independent lines carrying mutations in the *Hagoromo* locus develop NB-like tumors with high penetrance [92]. These fish carry viral insertions in the *fbxw4* gene that do not affect *fbxw4* expression levels but lead to a sustained and aberrant expression of *fgf8* after embryogenesis. Notably, tumors arising in *Hagoromo* mutants are quite different from human NB, since malignant masses develop in cranial ganglia and head of mutant fish. Moreover, tumor cells have a neuronal origin (Hu-positive) but do not express markers of catecholaminergic differentiation, such as *TH* or *DBH*, which indicates that these malignant cells originate from a different neural lineage than human NB. Despite these differences, *Hagoromo*-derived tumors seem to arise through a similar mechanism as in humans [92]. Thus, *fgf8* appears to function as an oncogene not only in numerous malignancies, including prostate and breast cancer in mammals [93, 94], but also in NB.

## Conclusions

Many cellular and molecular features of NB have been identified over the past decades, and some of these genetic aberrations are now considered strong and powerful predictors of patient outcome. Nevertheless, there is still more complexity behind this pediatric neoplasm, and continued advances are needed to translate this information into more efficient clinical therapy. Although murine and avian models have been used to dissect PSNS development and NB pathogenesis [95], zebrafish displays several advantages, including embryonic optical transparency, small size and external development, making it easier to follow NCC migration and differentiation. Importantly, the generation of new zebrafish models of NB can contribute to the knowledge of NB biology and genetics. Therefore, the generation and use of zebrafish transgenic and mutant lines may provide new opportunities to better understand the biology of NB and to test in vivo potential chemical tumor suppressors through high-throughput drug screening. In addition, zebrafish models of NB crossed with cell signaling reporter fish might be a powerful tool for the in vivo dissection of the signaling cascades and molecular mechanisms involved in tumor development [96, 97].

## Abbreviations

NB: neuroblastoma
PSNS: peripheral sympathetic nervous system
NCC: neural crest cell
OS: overall survival
CNV: copy number variation
ALK: anaplastic lymphoma kinase
EMT: epithelial to mesenchymal transition
hpf: hours post-fertilization
BMP: bone morphogenetic factor
dpf: days post-fertilization
SCG: superior cervical ganglion
TH: tyrosine hydroxylase
DBH: dopamine B-hydroxylase
